# Arterial blood pressure targets in septic shock: is it time to move to an individualized approach?

**DOI:** 10.1186/s13054-015-0958-x

**Published:** 2015-06-18

**Authors:** Thiago Domingos Corrêa, Stephan Matthias Jakob, Jukka Takala

**Affiliations:** Intensive Care Unit, Hospital Israelita Albert Einstein, Av. Albert Einstein, 627/701, São Paulo, 05652-000 Brazil; Department of Intensive Care Medicine, Inselspital, Bern University Hospital and University of Bern, Freiburgstrasse 10, CH-3010 Bern, Switzerland

## Abstract

Xu and colleagues evaluated the impact of increasing mean arterial blood pressure levels through norepinephrine administration on systemic hemodynamics, tissue perfusion, and sublingual microcirculation of septic shock patients with chronic hypertension. The authors concluded that, although increasing arterial blood pressure improved sublingual microcirculation parameters, no concomitant improvement in systemic tissue perfusion indicators was found. Here, we discuss why resuscitation targets may need to be individualized, taking into account the patient’s baseline condition, and present directions for future research in this field.

## Commentary

In a recent article in *Critical Care*, Xu and colleagues [1] evaluated the impact of increasing mean arterial blood pressure (MAP) levels from around 70 mmHg, through norepinephrine administration, on systemic hemodynamics, tissue perfusion, and sublingual microcirculation of 19 septic shock patients with chronic hypertension. The target MAP was defined as the patient’s average usual blood pressure in the previous 2 years. Increasing MAP improved sublingual microcirculation parameters without concomitant improvement in systemic tissue perfusion indicators (arterial pH, lactate, and urinary output) [1].

The concept of individualizing the MAP targets for patients with septic shock is certainly laudable. Several aspects of the study design and methodology should be considered in the interpretation of the results. First, patients were enrolled in a late phase of resuscitation, after an average of 16 h from the beginning of shock, with an unknown period of time spent with an MAP under 65 mmHg [1]. In experimental sepsis, increasing the delay between sepsis onset and the resuscitation maneuvers increases severity of sepsis and need for resuscitation [2]. Thus, one could argue that earlier microcirculation recruitment in such patients with hypertension would have been associated with better results in terms of tissue perfusion and outcomes. Second, the time between the baseline measurements and the second measurements at the patients’ usual MAP levels was only 50 min [1]. Any improvement in variables reflecting organ perfusion and metabolism is unlikely in such a short window of time if microcirculation in areas other than the sublingual region indeed did improve. Finally, as stressed by the authors, this was a single-center prospective open-label study without a concurrent control group. Therefore, it is not possible to rule out the role of chance (that is, the possibility that the observed improvement in microcirculatory parameters occurred by chance or as a consequence of the natural evolution of the disease).

In a recent large trial on septic shock, more than 40 % of patients had a history of chronic hypertension [3]. Patients with chronic hypertension usually need higher MAP levels than patients without hypertension to achieve and maintain an adequate perfusion pressure to the vital organs [4] and therefore their MAP goals may need to be revised [5].

Small prospective cohort studies [6–9] and two randomized studies [3, 10] addressed the impact of different MAP levels on tissue perfusion, organ function, sublingual microcirculation, and outcomes in patients with septic shock. The main findings of these studies were recently reviewed elsewhere [11]. The SEPSISPAM (Assessment of Two Levels of Arterial Pressure on Survival in Patients With Septic Shock Study) study included 776 patients with septic shock and demonstrated that higher MAP targets (80 to 85 mmHg) in comparison with conventional targets (65 to 70 mmHg) did not improve survival or the need for renal replacement therapy [3]. Of note, all patients in the lower target group were above the goal blood pressure range during the 5 protocol-specified days. Nevertheless, when the subgroup of patients with chronic hypertension was analyzed, targeting a higher MAP decreased the need for renal replacement therapy [3]. We recently demonstrated in a long-term porcine fecal peritonitis model that MAP targets between 75 to 85 mmHg compared with 50 to 60 mmHg did not improve global or regional hemodynamics but did decrease the incidence of acute kidney injury [12].

Taken together, the available evidence reveals that targeting higher MAP levels during the initial resuscitation of septic shock has variable effects on microcirculatory parameters and organ function, which may also be dependent on patients’ usual blood pressure level. However, it should be noted that targeting higher blood pressure increases patients’ exposure to fluids and vasopressors, which may have detrimental effects [13].

What do we learn from the study by Xu and colleagues? It is well known that patients admitted to the intensive care unit vary widely in terms of age, number and type of comorbidities, and functional status [14]. Such variability may explain, at least in part, the failure in translating many advances in basic science to clinical practice and why we have to face so many negative results in large sepsis multicenter randomized clinical trials [15]. Therefore, as proposed by Xu and colleagues, resuscitation targets may have to be individualized, taking into account the patient’s baseline condition [1].

Further research is needed to address the impact of individualized MAP targets on tissue perfusion, organ function, inflammatory response, and exposure to vasopressors and fluids and, more importantly, on the outcomes. The development of new technologies may help clinicians to evaluate, at the bedside, the impact of achieved MAP targets on microcirculatory parameters and organ function. Finally, the individualized approach may allow us to identify non-hypertensive patients who may tolerate lower mean arterial and perfusion pressure levels to maintain their organs’ function. In such patients, a decreased exposure to vasopressors may limit their side effects and improve outcomes.

## Abbreviations

MAP: Mean arterial blood pressure
